# Solving nucleic acid structures by molecular replacement: examples from group II intron studies

**DOI:** 10.1107/S0907444913013218

**Published:** 2013-10-12

**Authors:** Marco Marcia, Elisabeth Humphris-Narayanan, Kevin S. Keating, Srinivas Somarowthu, Kanagalaghatta Rajashankar, Anna Marie Pyle

**Affiliations:** a Department of Molecular, Cellular and Developmental Biology, Yale University, New Haven, CT 06511, USA; b The Northeastern Collaborative Access Team (NE-CAT), Advanced Photon Source, Argonne National Laboratory, Argonne, IL 60439, USA; c Department of Chemistry, Yale University, New Haven, CT 06511, USA; d Howard Hughes Medical Institute, Chevy Chase, MD 20815, USA

**Keywords:** nucleic acid sequence homology, *de novo* structure design, long noncoding RNA, RNA structure, homology modeling, *RCrane*

## Abstract

Structured RNA molecules are key players in ensuring cellular viability. It is now emerging that, like proteins, the functions of many nucleic acids are dictated by their tertiary folds. At the same time, the number of known crystal structures of nucleic acids is also increasing rapidly. In this context, molecular replacement will become an increasingly useful technique for phasing nucleic acid crystallographic data in the near future. Here, strategies to select, create and refine molecular-replacement search models for nucleic acids are discussed. Using examples taken primarily from research on group II introns, it is shown that nucleic acids are amenable to different and potentially more flexible and sophisticated molecular-replacement searches than proteins. These observations specifically aim to encourage future crystallographic studies on the newly discovered repertoire of noncoding transcripts.

## Abstract 2

Strategies for phasing nucleic acid structures by molecular replacement, using both experimental and *de novo* designed models, are discussed.

## 1. Introduction

The crystallographic phase problem (Muirhead & Perutz, 1963 ▶) can be solved using different approaches, of which molecular replacement (MR) has been the most widely used since its development in the early 1960s (Hoppe, 1957 ▶; Rossmann & Blow, 1962 ▶; Huber, 1965 ▶). Particularly today, MR can be broadly applied given that the number of structures, and thus of potential search models, is increasing exponentially in the open-access data banks (Rossmann, 2001 ▶). As of 19 February 2013, statistics run on the Protein Data Bank (PDB) show that of the almost 80 000 deposited macromolecular X-ray structures, about 60% were solved by MR.

In addition to the abundance of search models, the popularity of MR is also a consequence of the flexibility of this method, which is applicable to solving the structures of any type of macromolecule ranging from proteins to nucleic acids and their complexes. However, proportionally more protein than nucleic acid X-ray structures have currently been solved by MR. Why is there a discrepancy between these two classes of macromolecules? Do these numbers indicate that nucleic acids are intrinsically less amenable to structure determination by MR? A number of considerations need to be made in regard to this issue.

**Figure 1:**
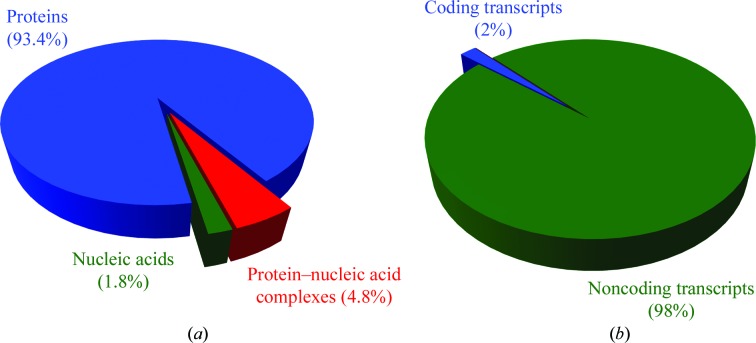
Statistics for nucleic acid *versus* protein structures. (*a*) Pie chart describing the percentage of X-ray structures solved containing different types of macromolecules. Nucleic acids are significantly underrepresented relative to proteins. Statistics were calculated from the PDB as of 19 February 2013. (*b*) Distribution of coding *versus* noncoding transcripts in the cell, as derived from genomic data analysis (Washietl *et al.*, 2007 ▶; The Encode Project Consortium, 2012 ▶). An increasingly large number of noncoding elements have been shown to possess distinct tertiary structures; therefore, the quantity and the variety of nucleic acid crystallography targets are rapidly increasing.

Interestingly, the first protein and nucleic acid structures determined by MR were obtained at approximately the same time. Specifically, the structures of the *Tobacco mosaic virus* capsid protein (Jack, 1973 ▶) and of glyceraldehyde-3-phosphate dehydrogenase (Argos *et al.*, 1975 ▶) were determined only shortly before the structure of tRNA^fMet^ (Woo *et al.*, 1980 ▶). However, the rate of structure determination did not proceed at the same pace for the two classes of macromolecules. Today, nucleic acid X-ray structures represent only 1.8% of the total number of structures available in the PDB, and the structures of protein–nucleic acid complexes make up 4.8% of the total. In contrast, protein-only structures account for the remaining 93.4% (Fig. 1 ▶). With so few potential search models, it is not surprising that a smaller percentage of nucleic acid structures have been solved by MR.

Nucleic acid structure determination lags behind protein structure determination primarily because it has only recently been discovered that there are a wealth of structured non­coding RNA elements in cells (Washietl *et al.*, 2007 ▶; The Encode Project Consortium, 2012 ▶; Fig. 1 ▶). Certainly, there is no theoretical or practical preclusion for the success of crystallographic studies on nucleic acids. In fact, the physics underlying crystallization, the geometry of the diffraction experiment and the choices of the methods used for structure determination are essentially the same for both nucleic acids and proteins (Scott, 2012 ▶). Moreover, the vast majority of software currently used for data analysis and structure determination can process nucleic acid structures as efficiently as protein structures. However, in comparison to protein crystallography, nucleic acid crystallography does present certain technical and practical challenges (Baikalov & Dickerson, 1998 ▶). These differences strongly affect the choice and the design of MR search models, which is the most crucial step in obtaining correct structure solutions (Evans & McCoy, 2008 ▶). Therefore, in this report we discuss the preparation of search models for use in solving nucleic acid structures by MR. Specifically, we present examples and test cases related to our research on the structure of group II introns, which are 400–­1000-nucleotide noncoding RNAs that are capable of self-splicing and retrotransposition (Pyle, 2010 ▶).

### 1.1. Properties of nucleic acids

Just like proteins, nucleic acids possess distinctive features at the primary, secondary and tertiary structure levels. These distinctions influence not only the function of these macromolecules but also their structural organization. Taking these properties into account is important when developing software to determine, analyze and model the three-dimensional structures of nucleic acids. Therefore, consideration of these differences is also critical when solving nucleic acid structures *via* MR.

At the sequence level, nucleic acids are composed of simpler building blocks than proteins. Their sequence is less variegated, as it consists of a combination of only four nucleobases rather than the 20 amino acids that compose protein chains. As discussed below, this property makes nucleic acid structure less sensitive to differences in sequence, which is advantageous when applying the MR approach to nucleic acids.

Also, the secondary structure of nucleic acids, which refers to the helices typical of DNA and RNA (Moore, 1999 ▶), is simpler than that of proteins. DNA generally forms helical structures of the B-form (or, occasionally, the A-form or Z-­form), while RNA molecules are primarily built from combinations of A-form helices (Scott, 2012 ▶). All of these motifs have a well known conserved geometry, so that (with some exceptions) their secondary structure can be predicted with a higher degree of confidence than protein secondary structure (Baikalov & Dickerson, 1998 ▶). The high internal symmetry of nucleic acid helices adds nontrivial complications to the MR search functions (Baikalov & Dickerson, 1998 ▶). However, in some cases, such symmetry may constitute an advantage for the MR approach by allowing structure solution even in the absence of experimental models (see below).

Despite the simplicity of their sequences and secondary structures, nucleic acids adopt intricate tertiary architectures and a wide variety of recurring three-dimensional motifs. The elaborate architecture of RNA is apparent from recent structures of ribosomes, riboswitches, self-splicing introns and many other RNAs (Butcher & Pyle, 2011 ▶). The identification of RNA tertiary motifs is enriching our understanding of the nucleic acid structural space and it will allow the compilation of useful ‘libraries’ of nucleic acid structural building blocks. The latter will be useful in the future for identifying appropriate search models for MR.

Finally, the length of nucleic acid molecules is an important factor. In this report, we divide nucleic acids into three classes. Nucleic acids of up to 50 bases are defined as small, those of 50–200 nucleotides in length as medium and those with more than 200 nucleotides as long. Each of these three groups possesses properties that favor or hamper the MR approach to differing extents (Table 1 ▶).

### 1.2. Choice and design of suitable MR models for nucleic acids

As mentioned above, the most crucial step in solving structures of macromolecules by MR is the identification of a good search model. Such a model is characterized by high structural similarity to the target [*i.e.* a root-mean-square deviation (r.m.s.d.) of <2.5 Å; Evans & McCoy, 2008 ▶]. In most cases the structural similarity between the model and target cannot be determined accurately *a priori*, so MR typically requires a trial-and-error process (Evans & McCoy, 2008 ▶). However, in general the coordinates of the model can be (i) derived from available experimental data, such as other X-ray or nuclear magnetic resonance (NMR) structures (Jung & Zweckstetter, 2004 ▶; Langmead *et al.*, 2004 ▶; Mao *et al.*, 2011 ▶; Thompson *et al.*, 2012 ▶), or (ii) created in the absence of experimental data (Claude *et al.*, 2004 ▶; Giorgetti *et al.*, 2005 ▶; DiMaio *et al.*, 2011 ▶). In the former case, the model can be represented (i) by a homologous structure, (ii) by the structure of the target molecule that is itself in a different state, space group or conformation or (iii) by the structures of domains of (homologues of) the target molecule. In the latter case, the model can instead be designed (iv) by homology modeling (Giorgetti *et al.*, 2005 ▶) or (v) by *de novo* structure predictions (Thompson & Baker, 2011 ▶). Recently, thanks to new software that randomly searches the accessible data banks, broader less user-biased trials can also be performed, thus enhancing the chances of obtaining interpretable MR solutions (Stokes-Rees & Sliz, 2010 ▶). Finally, the input of weak experimental phases determined by anomalous scattering can enhance the chances of success of the MR search (Kleywegt & Jones, 1997 ▶).

In the following paragraphs, we will describe these different strategies applied to nucleic acids and we will support our statements with examples from our own research and that of other investigators.

## 2. MR searches in the presence of experimental models

### 2.1. Structural homology among nucleic acids

In protein crystallography, a sequence identity of 30–35% to the target is generally accepted as a criterion for selecting MR search models (Chothia & Lesk, 1986 ▶; Claude *et al.*, 2004 ▶; DiMaio *et al.*, 2011 ▶). This cutoff can be lowered further if the alignment between model and the target is highly accurate (Schwarzenbacher *et al.*, 2004 ▶).

For nucleic acids, the sequence-to-structure correlations are less well understood than for proteins and current statistics suggest that they are weaker (Capriotti & Marti-Renom, 2010 ▶). It has been proposed that short RNA loops need to share a sequence identity of 75% to infer structural similarity with confidence (Schudoma *et al.*, 2010 ▶) and this increases to 85% in paralogous mRNA pairs (Chursov *et al.*, 2012 ▶). At the same time, many noncoding RNA molecules possess well conserved secondary and tertiary folds but share low sequence similarity (Dirheimer *et al.*, 1995 ▶; Zhang *et al.*, 2005 ▶; Yu, 2011 ▶). Therefore, identifying suitable structural homologues for new nucleic acid targets may be challenging using only sequence-similarity software, *i.e.* *BLAST* (Altschul *et al.*, 1990 ▶), *MUSCLE* (Edgar, 2004 ▶), *T-Coffee* (Notredame *et al.*, 2000 ▶) or *ClustalW* (Thompson *et al.*, 1994 ▶).

This limitation may be overcome by considering the fact that nucleic acids possess greater evolutionary flexibility than proteins. Indeed, nucleic acid helices may possess identical structures even if their sequences differ dramatically, provided that the base pairing between the partner strands is maintained. Identifying evolutionary covariations in the sequences of partner strands is possible through comparative sequence analysis (Fox & Woese, 1975 ▶; Pace *et al.*, 1989 ▶; Woese & Pace, 1993 ▶). This method can help to identify clusters of homologous molecules that do not share significant sequence conservation but possess common secondary-structure motifs and thus potentially similar tertiary structures. Therefore, structural similarity rather than sequence-similarity search algorithms need to be used to identify homologues of nucleic acids, *i.e.* *CMfinder* (Yao *et al.*, 2006 ▶), *Infernal* (Nawrocki *et al.*, 2009 ▶) or *LocARNA* (Will *et al.*, 2007 ▶), or software performing whole-genome alignment (Yu, 2011 ▶).

### 2.2. MR searches on nucleic acids are not sensitive to sequence conservation

Since nucleic acids may possess homologous structures even if they share very low sequence identity, we investigated the sensitivity of MR searches to nucleic acid sequence divergence using data sets and structures obtained in our laboratory for the *Oceanobacillus iheyensis* group II intron (Toor *et al.*, 2008 ▶; Marcia & Pyle, 2012 ▶). Briefly, group II introns are a broad class of self-splicing RNA molecules that are essential for gene expression in many organisms (Pyle & Lambowitz, 2006 ▶). They constitute a good system both to exemplify sequence covariation in RNA and to study the sensitivity of MR programs to sequence divergence, mainly because all group II introns are expected to possess a common structural fold (Michel *et al.*, 2009 ▶; Toor *et al.*, 2009 ▶) despite the sequences of different group II intron subtypes having diverged significantly (Michel *et al.*, 1989 ▶; Michel & Costa, 1998 ▶).

**Figure 2:**
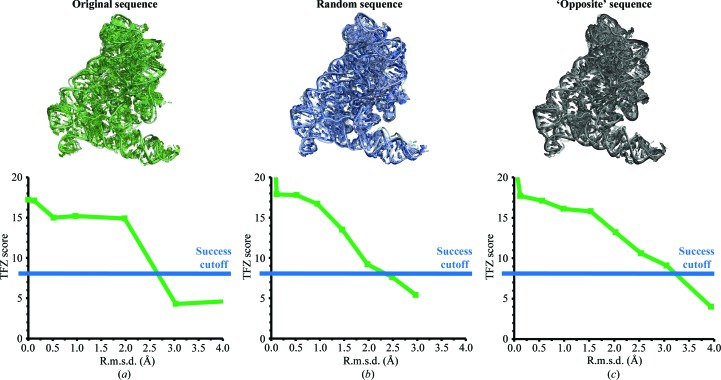
The low sensitivity of MR searches to nucleic acid sequence conservation. Group II intron structures were derived from the *O. iheyensis* group II intron structure (PDB entry 3igi) using (*a*) the original intron sequence (green tones), (*b*) a randomly generated sequence (blue tones) or (*c*) an ‘opposite’ sequence (gray tones). For each sequence, the backbone was distorted using *FRODA* with increasing values of the r.m.s.d. (from 0 Å in the darkest color to 4 Å in the lightest color). The TFZ scores from *Phaser* are plotted for each MR run. A TFZ value of 8 or higher was observed to confidently indicate correct solutions for protein structures (McCoy, 2007 ▶) and is thus taken here as a cutoff level for successful solutions. Although such a TFZ scale may not necessarily apply exactly to nucleic acid structures, we observed that our solutions with TFZ > 8 were generally associated with interpretable electron-density maps.

For our investigation, we generated two random RNA sequences of 390 nucleotides in length (corresponding to the length of the crystallized form of the *O. iheyensis* group II intron). In one case, we used the *Random DNA Sequence Generator* software (http://www.faculty.ucr.edu/~mmaduro/random.htm), obtaining a sequence with 21% identity to the *O. iheyensis* group II intron. In the other case, we artificially designed a completely new sequence with no identity to the *O. iheyensis* group II intron, mutating all purines to pyrimidines and *vice versa*, thus bringing the sequence divergence to an extreme case. We used these artificial sequences to modify the identity of the nucleobases in a structure of the *O. iheyensis* group II intron (PDB entry 3igi; Toor *et al.*, 2010 ▶) using *Coot* (Emsley *et al.*, 2010 ▶), thereby changing the primary structure but not the secondary and tertiary structures of 3igi. Finally, for each case we created a series of MR search models by exploring the dynamics of the RNA backbone in the range of r.m.s.d. values from 0 to 4 Å using normal-mode analysis in *FRODA* (Fulle & Gohlke, 2008 ▶). Phasing attempts in *Phaser* (McCoy, 2007 ▶) using the experimental structure-factor amplitudes of the structure with PDB code 4faw (Marcia & Pyle, 2012 ▶) were successful with both sets of models, having r.m.s.d. values of up to 2–3 Å from the original (Fig. 2 ▶). As expected, these results show that MR searches for nucleic acids are not affected by sequence divergence, only by the structural similarity between search model and target.

Based on the considerations above, we conclude that structure-similarity searches accounting for sequence covariation are necessary to identify nucleic acid homologues and we suggest that the low level of sequence conservation of such homologues need not limit their use as search models for MR approaches.

### 2.3. Minimal MR search models for nucleic acids

**Figure 3:**
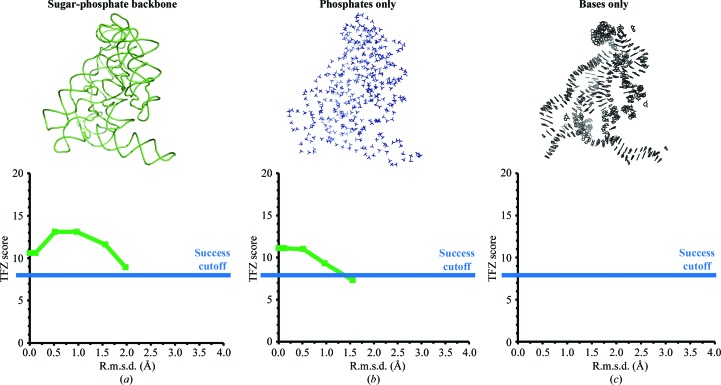
Minimal MR models for nucleic acid structures. The structure of the group II intron can be successfully solved (TFZ > 8 in *Phaser* and interpretable electron-density maps) using only the sugar-phosphate backbone distorted by up to 2 Å r.m.s.d. (*a*) or only the phosphate groups distorted by up to 1 Å r.m.s.d. (*b*) but not by using only the coordinates of the nucleobases (no *Phaser* solution was obtained) (*c*).

Two factors explain the low sensitivity of MR programs to sequence divergence in nucleic acids, namely the high structural similarity of the four nucleobases and the fact that the nucleobases contribute only minimally to the total X-ray scattering. Rather than the identity of the nucleobases, the position of the sugar-phosphate backbone of nucleic acids is a much stronger determinant for the success of MR searches. Indeed, MR search models of nucleic acids can be pruned by removing all nucleobases, and in some cases also the sugar moieties, without affecting the success of the phasing process. To exemplify the latter concept, we again used the group II intron data as a test case. From a series of structures obtained with *FRODA* from PDB entry 3igi and characterized by increasing r.m.s.d. values (0–4 Å), we derived substructures removing either all of the nucleobase atoms, the nucleobase and sugar moieties or the sugar and phosphate groups. We then performed MR runs in *Phaser*, attempting to phase the experimental structure-factor amplitudes of PDB entry 4faw. We determined that the sugar–phosphate backbone (with r.m.s.d. values of up to 2 Å relative to the original structure) and even the phosphate groups by themselves (with r.m.s.d. values of up to 1 Å) are sufficiently good starting models to obtain correct solutions in *Phaser*, with TFZ scores greater than 8 and interpretable output electron-density maps. However, the bases alone do not possess sufficient scattering information for the MR software to phase the target data set correctly, regardless of the r.m.s.d. (Fig. 3 ▶).

These tests suggest that it is possible to use minimal MR models composed only of backbone elements. This is similar to what is performed in protein structure determination with the use of polyalanine (DeLano & Brünger, 1995 ▶), polyglycine (Fabiane *et al.*, 1998 ▶; Hausrath *et al.*, 1999 ▶) or polyserine (Storici *et al.*, 1999 ▶; Minor *et al.*, 2000 ▶) models.

### 2.4. Isomorphism of nucleic acid structures

Another common case for MR is the study of targets whose structures are known but for which new data have been obtained, *i.e.* in novel crystallization conditions, space groups or functional states. For such situations, 100% sequence conservation is not a guarantee of structural identity (Kosloff & Kolodny, 2008 ▶) and MR may still be unsuccessful if the structural similarity is not maintained. Indeed, strong structural differences of >6 Å r.m.s.d. between pairs of identical targets can be caused by the binding of ligands or cofactors, by the influence of solvent molecules, by the adoption of alternative conformations, by the establishment of alternative quaternary-structure interactions or by domain swapping (Liu & Eisenberg, 2002 ▶; Kosloff & Kolodny, 2008 ▶).

Moreover, certain amino-acid sequences adopt different secondary structures depending on their molecular environment, as exemplified by moonlighting proteins (Jeffery, 1999 ▶) and promiscuous enzymes (Khersonsky *et al.*, 2006 ▶). The same considerations are believed to affect nucleic acids considerably more frequently than proteins. For instance, the structure of group II intron domain 5 (D5) in isolation is significantly different from that in the context of the intact enzyme (Zhang & Doudna, 2002 ▶; Sigel *et al.*, 2004 ▶; Toor *et al.*, 2008 ▶). Such behavior can be attributable to two main reasons. Firstly, the free-energy landscape of nucleic acid molecules is flatter than that of proteins, implying that a given nucleic acid sequence may possess a large number of favored conformations (Zuker, 1989 ▶). It has been estimated that RNA molecules of *N* nucleotides can fold into about 1.8^*N*^ based-paired secondary structures with many potential tertiary folds (Zuker & Sankoff, 1984 ▶; Hajdin *et al.*, 2010 ▶). Secondly, nucleic acid structures may be more dependent on solvent molecules and ions than proteins. For example, metals determine and stabilize the structure of certain nucleic acid motifs (Butcher & Pyle, 2011 ▶), such as the kink turn (Goody *et al.*, 2004 ▶), and they also modulate nucleic acid flexibility (Al-Hashimi *et al.*, 2003 ▶). As further evidence of the importance of metal ions in nucleic acids, the three-dimensional X-ray structures of these macromolecules contain an average of approximately 15 metal ligands (Stefan *et al.*, 2006 ▶; Schnabl *et al.*, 2012 ▶), while metallo­proteins typically contain only 1–5 (Nakamura *et al.*, 2009 ▶). As a result, small changes in solvent molecules can cause very pronounced non-isomorphism in nucleic acid structures. This non-isomorphism can lead to difficulties in MR and similarly affects heavy-atom replacement techniques such as single/multiple isomorphous replacement (SIR/MIR; Baikalov & Dickerson, 1998 ▶).

However, we recently reported a rather surprising result showing that a nucleic acid molecule can be far more accommodating to different solvent conditions than is generally expected. During our work on the *O. iheyensis* group II intron, we were able to crystallize our target with a wide variety of metal-ion combinations, including alkali metals (Li^+^, Na^+^, K^+^, Rb^+^ and Cs^+^), alkaline-earth metals (Mg^2+^ and Ba^2+^) and post-transition metals (Tl^+^), and with non-metallic ions (NH_4_^+^) (Marcia & Pyle, 2012 ▶). While other research groups used transition metals in combination with physiological ions (*i.e.* K^+^ and Mg^2+^) to characterize their targets (Stahley *et al.*, 2007 ▶), with the group II intron we were successful in obtaining well diffracting crystals when we fully replaced the physio­logical metals K^+^ and Mg^2+^ with analogues directly in the crystallization buffer. The resulting crystal structures revealed a surprising degree of adaptation in accommodating all of these ions (Marcia & Pyle, 2012 ▶). We obtained a total of 14 different structures, all isomorphous to each other (r.m.s.d. of ∼1 Å), and we could efficiently solve all of them using MR. These structures allowed us to detect conformational changes in the intron and to visualize different intermediate catalytic forms of this ribozyme, which allowed an improved understanding of the catalytic mechanism (Marcia & Pyle, 2012 ▶). Additionally, the identification of these new features confirms that our structures did not suffer from model bias, which is a matter of great concern during MR experiments, especially when using models that are very similar to the targets (Kleywegt, 2000 ▶; Terwilliger, 2004 ▶). In summary, our work shows that large noncoding RNAs can indeed maintain the same fold in the presence of nonphysiological ions. This allows metal soaks and MR to serve as a valuable tool for determining and interpreting the structural and functional properties of nucleic acids.

### 2.5. Multi-domain MR searches for nucleic acids

In the previous sections, we described the use of the entire *O. iheyensis* group II intron structure as a single search model in MR searches. However, typical MR pipelines do not account for rigid-body motions amongst the different domains that compose three-dimensional structures. Therefore, these pipelines may fail if the individual domains are not used as separated search ensembles (Cygler & Anderson, 1988*a* ▶,*b* ▶). For multi-domain proteins, important factors for the success of MR are the completeness of the composite model, the size and number of the individual domains and, in some cases, the order in which the domains are used in the iterative MR search (Evans & McCoy, 2008 ▶; Luo *et al.*, 2011 ▶).

Here, we discuss some facts to consider when generating domain ensembles for nucleic acids. The group II intron can provide an illustrative example, as it is composed of six domains (Pyle, 2010 ▶). Briefly, domain 1 (D1; 265 nucleotides long in *O. iheyensis*) is the most intricate and is composed of six smaller subdomains (D1i-ii, D1a, D1b, D1c, D1d1 and D1d2). Each subdomain primarily consists of a relatively simple and short helical fragment (Pyle, 2010 ▶). The other domains protrude out from the D1 structural core and are smaller in size. In the *O. iheyensis* group II intron construct engineered in our laboratory for crystallization purposes (Toor *et al.*, 2008 ▶), these other domains possess the following characteristics. Domain 2 (D2) is formed by a single eight-base-pair stem terminating in a tetraloop (20 nucleotides in total). Domains 3 and 4 (D3 and D4) both contain two stems separated by a bulged region, with D3 longer than D4 (38 and 27 nucleotides, respectively). D5 is 34 nucleotides long and has a characteristic ‘elbow’ shape (Pyle, 2010 ▶). The tertiary structure of D6 is still undetermined (Pyle, 2010 ▶).

We considered two strategies to create individual domain ensembles for the group II intron. We first followed a strategy that is typical in protein crystallography. We used ensembles covering each of the five domains from a previously published intron structure (PDB entry 3igi; Toor *et al.*, 2008 ▶). We implemented this strategy using *Phaser* (McCoy, 2007 ▶) and a more recent data set (PDB entry 4faw; Marcia & Pyle, 2012 ▶). The MR search was quick and it converged to a solution characterized by high scores for the three larger domains (TFZ = 57.8 for D1, TFZ = 33.9 for D3 and TFZ = 42.8 for D5). These results provided an interpretable electron-density map suitable for further structural refinement. Only the two smaller domains of the intron (D2 and D4) were placed incorrectly in the unit cell. This test suggests that when the structures of individual domains are known and are maintained in the context of the entire molecule it is possible to perform nucleic acid multi-domain searches successfully, similar to the searches performed for protein structures.

**Figure 4:**
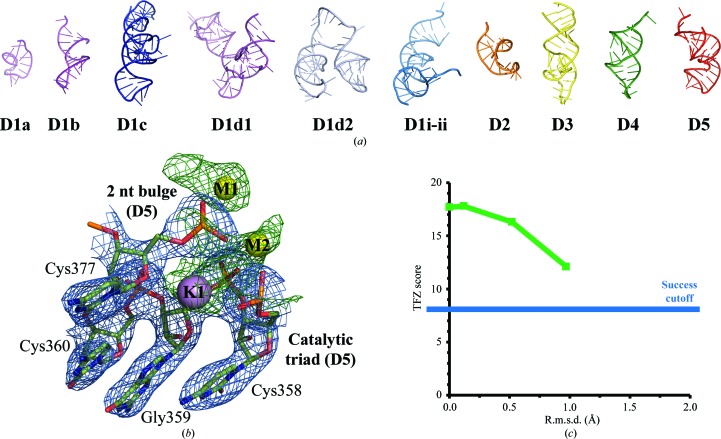
Multi-domain MR searches for phasing nucleic acid structures. (*a*) The experimental structure-factor amplitudes of an *O. iheyensis* group II intron data set (PDB entry 4faw) can be phased with a *Phaser* multi-domain MR search using ten individual intron subdomains as a starting model. (*b*) The resulting σ-weighted 2*F*_o_ − *F*_c_ electron-density map is shown around the active-site motifs in blue mesh (1.5σ contour level). The positive signal in the σ-weighted *F*_o_ − *F*_c_ map (green mesh, 3.0σ) is shown at the expected position of the catalytic metal ions, which were not included in the search model (M1/M2 and K1; yellow and purple spheres, respectively). (*c*) Successful solutions (TFZ > 8) could be obtained using intron subdomain structures distorted by up to 1 Å r.m.s.d. with respect to their original structure in the model (PDB entry 3igi).

Our second approach to solving the group II intron structure *via* MR used individual short helical fragments as independent ensembles. We first removed junction nucleotides from the search structure (PDB entry 3igi) and then divided the structure into ten helical fragments, each representing a single subdomain, including each separate subdomain of D1. Together, these helices covered approximately 85% of the intron structure. The helices varied in length, but many were structurally similar, with reciprocal r.m.s.d.s of approximately 4 Å or less. Thus, we expected an MR search to be complex and unsuccessful. However, *Phaser* was able to model eight of the ten helical fragments into the structure, resulting in a high score (TFZ = 17.7) and an interpretable electron-density map (Fig. 4 ▶). Interestingly, successful solutions were also obtained when the backbone of the individual subdomains used in the search were distorted by normal-mode analysis in *FRODA* by up to 1 Å r.m.s.d. with respect to their original structure in PDB entry 3igi (Fig. 4 ▶).

These results are encouraging because they suggest that small helical fragments can be a powerful tool for solving large nucleic acid structures, rather than confining the technique to small and medium-sized molecules (Robertson *et al.*, 2010 ▶). Given our results above, we currently believe that a maximum of 6–8 helical fragments, each not shorter than 12–15 base pairs, could be used as MR search models without making the MR search too long or too complex. These numbers may increase if more elaborate models possessing distinctive folds are designed, *i.e.* models containing bulges, loops and junctions. These considerations point towards the potential of exploiting *de novo* modeling tools that can build a variety of fragments for use as MR search models (Humphris-Narayanan & Pyle, 2012 ▶; see below).

## 3. Phased-MR approaches

**Figure 5:**
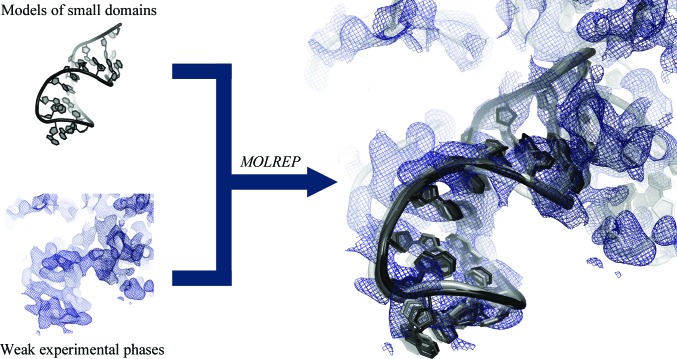
Phased MR. The D2 domain of the group II intron (black cartoon diagram; top left panel) was not placed correctly in multi-domain MR searches (see Fig. 4 ▶). However, it can be assigned to its expected position in the electron density (right panel) by *MOLREP* if information from weak experimental phases is provided (bottom left panel). The solvent-flattened experimental electron-density map is depicted as a blue mesh at a 1.5σ contour level.

In our multi-domain structural search using the ten intron subdomains, we noticed that *Phaser* encountered problems in placing two domains (D2 and D4). These domains possess less distinct tertiary motifs and are the shortest domains in the structure. To aid in the assignment of such small domains, information from weak experimental phases could potentially support and enhance the MR solution (Kleywegt & Jones, 1997 ▶). Therefore, we attempted to identify the correct positions of D2 and D4 using phased MR. This analysis involved the structures of D2 and D4 extracted from 3igi along with poor experimental phases obtained from a data set for the group II intron cocrystallized with Yb^3+^ (Toor *et al.*, 2008 ▶). The phase information provided by this single data set was insufficient to produce a traceable electron-density map (Toor *et al.*, 2008 ▶), but it enabled us to place both D2 and D4 correctly using *MOLREP* (Vagin & Teplyakov, 2010 ▶). This process was also successful when using D2 and D4 structures that had been distorted by up to 1 Å r.m.s.d. in *FRODA* (Fig. 5 ▶). Notably, we obtained successful *MOLREP* solutions by excluding higher resolution shells from the calculation, possibly because the phase information for these shells is of lower accuracy than the low-resolution and intermediate-resolution shells.

The phased-MR approach can also be run iteratively, where it places the different domains of the intron into the asymmetric unit one after another. This potentially enhances the probability of identifying the position of smaller domains. However, this strategy does not significantly help the difficult process of placing domains whose structures differ greatly from the target molecule. For instance, attempts to position D2 or D4 models with greater than 1 Å r.m.s.d. distortion were unsuccessful even after positioning D1, D3 and D5.

Thus, experimental phases and MR can be used synergistically. Heavy-atom soaking can be used successfully in nucleic acid crystallography (see §[Sec sec2.4]2.4) but it often yields experimental phases of poor quality (Toor *et al.*, 2008 ▶). Combining this information with MR searches significantly increases the success of structure solution.

## 4. MR searches in the absence of experimental models

Traditionally, fragments and domains from previously published structures are used to create MR search models. However, polypeptide fragments created using *in silico* design are becoming increasingly common MR search models thanks to the ever-growing richness of the structural databases, the availability of computational power and the improved accuracy of prediction algorithms (Gubbi *et al.*, 2007 ▶). Similarly, polynucleotide structures computed *in silico* can also be used as search models for MR of nucleic acids. To date, idealized helical fragments have been used for the study of short DNA chains (Baikalov & Dickerson, 1998 ▶) and of RNA molecules of medium size, such as the 70-nucleotide L1-ligase ribozyme (Robertson & Scott, 2007 ▶; Robertson *et al.*, 2010 ▶; Scott, 2012 ▶). While in certain cases helices with ideal geometry may also be used in the study of large nucleic acids, structure solution of the latter significantly benefits from the use of more sophisticated search models, *i.e.* models generated by homology modeling or *de novo* design.

### 4.1. Homology modeling of nucleic acid structures

Homology modeling is the most successful structure-prediction method for proteins and it is routinely used to guide various experimental studies (Martí-Renom *et al.*, 2000 ▶; Lukk *et al.*, 2012 ▶). Thanks to the efforts of CASP (Critical Assessment of Structure Prediction), fully automated methods are available for modeling proteins with respectable accuracy (Kryshtafovych *et al.*, 2011 ▶; Mariani *et al.*, 2011 ▶). In addition, there are databases of three-dimensional models of proteins for which the structure of at least one homolog is known and deposited in the PDB (Pieper *et al.*, 2004 ▶). However, very few methods are available for performing homology modeling on nucleic acids (Rother, Rother, Boniecki *et al.*, 2011 ▶). This is largely owing to the difficulty in identifying structural homologs. As mentioned above, there are far fewer nucleic acid structures available than there are for proteins, and nucleic acid homology is difficult to detect using only sequence data. Methods that incorporate secondary-structure data, such as *FASTR*3*D* (Lai *et al.*, 2009 ▶), *RNAFRABASE* (Popenda *et al.*, 2008 ▶) and *FR*3*D* (Sarver *et al.*, 2008 ▶), are more successful in identifying nucleic acid templates. Furthermore, an accurate alignment between the target and the template is also critical for homology modeling. Rather than sequence-based alignments, secondary-structure-based alignment methods such as *R-Coffee* (Moretti *et al.*, 2008 ▶) offer greater accuracy. Additionally, manual adjustment is frequently necessary to improve the quality of the alignment beyond what is currently possible using automated methods (Rother, Rother, Puton *et al.*, 2011 ▶). Once the template has been identified and aligned, tools such as *ModeRNA* (Rother, Rother, Puton *et al.*, 2011 ▶) or *RNAbuilder* (Flores & Altman, 2010 ▶; Flores *et al.*, 2010 ▶) allow the construction of a three-dimensional model.

### 4.2. *De novo* design of nucleic acid structural models

In addition to models constructed using homology, *de novo* modeling can also be used to aid MR in a number of ways. Small helices, tertiary motifs or potentially entire domains can be generated *de novo* and used as search fragments. Recently, an energy-based modeling protocol (*FARFAR*) was shown to model over half of 32 RNA motifs to within 1–2 Å r.m.s.d. accuracy (Das *et al.*, 2010 ▶). Thus, *de novo* generation of tertiary components for MR may currently be feasible. Further, in cases where a suitable homology model exists, *de novo* modeling can be used to refine regions that contain insertions or deletions. For example, exhaustively generating and evaluating RNA loop conformations can lead to accurate *de novo* models for small RNA loops (Sripakdeevong *et al.*, 2011 ▶).

**Figure 6:**
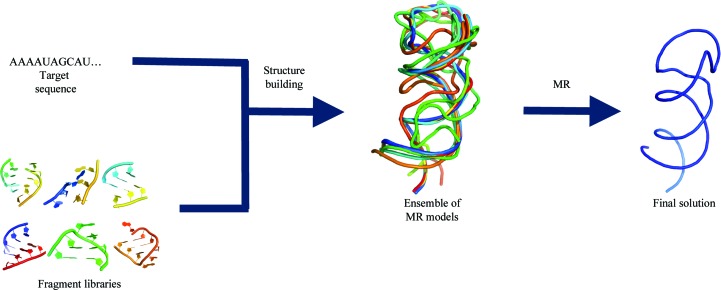
*De novo* design of nucleic acid domains to be used as MR search ensembles. Using a target sequence and a fragment library (left-hand panel), *de novo* design techniques can be used to build hundreds or thousands of models (middle panel). Each model could be scored based on an energy function and, if applicable, its accuracy of fit to an initial experimental density. Promising models could then be used as starting points for further tracing and refinement. Iterative model rebuilding and energy-guided optimization could significantly increase the quality of the final solution (right-hand panel, blue structure).

An additional and important use for modeling is to generate hundreds or even thousands of structural models *de novo* or from a starting homology model or backbone trace. ‘Core’ regions of a starting model that were highly conserved within a multiple sequence alignment could be modeled consistently among each ensemble member. Simultaneously, a *de novo* sampling or optimization protocol could be used to generate greater conformational variability in other regions. Strategies for introducing conformational variability include the sampling of alternative structures with an r.m.s.d. close to a starting structure (Humphris-Narayanan & Pyle, 2012 ▶) or the use of an energy-based sampling protocol such as *FARFAR* (Das *et al.*, 2010 ▶). A subset of low-energy *de novo* models can then be selected to solve the phase problem using MR. If MR using homology models or tertiary components has already generated density that is insufficient for all-atom autobuilding, this initial density could be utilized as an additional energy term to help select low-energy models. Once *de novo* models have been selected for MR, a new set of phases could be calculated to generate an improved electron-density map. Ensemble modeling and density-based refinement of homology models has been highly successful for MR in proteins (DiMaio *et al.*, 2011 ▶). Thus, a similar strategy of *de novo* model optimization and refinement may aid in solving RNA structures using MR in the future (Fig. 6 ▶).

## 5. Optimizing and refining MR models for nucleic acids with *RCrane*

Once a model has been chosen, its optimization and manual refinement may be beneficial and may increase the chances of success of the MR search. *RCrane* is a methodology for constructing and correcting RNA backbone structure (Keating & Pyle, 2010 ▶, 2012 ▶) and allows such model optimizations. This technique uses both the RNA pseudotorsions (Wadley *et al.*, 2007 ▶) and the RNA backbone conformers (Richardson *et al.*, 2008 ▶) to accurately predict and build all-atom RNA structures starting from only the phosphate and base positions. *RCrane* uses the θ′ and η′ pseudotorsions, where θ′ is defined as the P*_i_*—C1′*_i_*—P*_i_*_+1_—C1′_*i*−1_ dihedral and η′ is defined as the C1′_*i*−1_—P*_i_*—C1′*_i_*—P*_i_*_+1_ dihedral (Keating & Pyle, 2010 ▶). These pseudotorsions allow an accurate characterization of the RNA backbone using a coarse-grained representation. Conversely, the RNA backbone conformers enumerate roughly 50 detailed backbone configurations using the standard backbone torsions δ_−1_, ∊_−1_, ζ_−1_, α, β, γ and δ (Richardson *et al.*, 2008 ▶). *RCrane* uses the pseudotorsions and related coarse-grained information to predict the appropriate backbone conformer with high accuracy: one of the first three conformer predictions is correct 98% of the time and the first prediction is correct 84% of the time. Additionally, this technique is highly tolerant of errors in the phosphate and C1′ coordinates. Errors of up to 1 Å in the phosphate coordinate lead to only a 6% decrease in prediction accuracy, and errors of up to 2 Å in the C1′ coordinate lead to only a 13% decrease in prediction accuracy (Keating & Pyle, 2010 ▶). After conformer prediction, a multidimensional minimization procedure is used to build the remaining backbone coordinates.

*RCrane* is implemented as a plugin for *Coot* (Emsley *et al.*, 2010 ▶) and is frequently used for building RNA into low-resolution electron density solved through traditional phasing approaches such as SAD or MAD. However, the technique is equally applicable to structures solved using MR. As discussed above, search models may be constructed using homology modeling. The techniques used for homology modeling frequently build structures that match the overall architecture of the RNA, but the detailed backbone structure contains numerous steric clashes and improper backbone configurations. These issues can lead to improper placement of phosphates, which are particularly important in MR owing to their scattering power. Because of the high error tolerance of the *RCrane* predictions, the technique is still able to accurately determine the appropriate backbone conformer despite these improper phosphate placements. The minimization procedure used for building the backbone conformer can then also be used to correct the placement of the phosphate atoms, even in the absence of an electron-density map. These corrections allow *RCrane* to notably improve the phasing power of the MR search model.

*RCrane* is also applicable after the structure has been phased. In *RCrane* 1.1 or newer, the extend-chain option can be used to build missing portions of the model, as it allows the crystallographer to build onto the end of an existing RNA segment. This feature is particularly useful when phasing *via* MR, as the replacement model frequently does not cover all of the crystallized nucleotides. Additionally, *RCrane* can be used to correct portions of the model that do not match the newly phased electron density. Nucleotides that need minor adjustments can be corrected *via* rotamerization, which uses the existing phosphate and base positions to rebuild the backbone structure. Regions of the structure that need more dramatic corrections can be rebuilt from scratch using the extend-chain option. These correction procedures can continue to be used after crystallographic refinement to further improve the model (Keating & Pyle, 2010 ▶, 2012 ▶).

## 6. Conclusions and future perspectives

In this report, we have examined structure determination using MR and highlighted the differences in this technique when solving nucleic acid rather than protein structures, with a particular emphasis on the selection and design of the search model. These steps are generally the most crucial in determining the success of MR approaches (Evans & McCoy, 2008 ▶) and we envisage that they will become particularly important in the future for MR of nucleic acids. While the number of available nucleic acid structures does not yet match the number of protein structures, nucleic acid crystallography is rapidly expanding. Highly structured nucleic acids are now known to be essential players in an extensive variety of biological processes. Additionally, 98% of the transcriptome consists of noncoding transcripts (The Encode Project Consortium, 2007 ▶), and a large fraction of these are expected to adopt well defined stable tertiary structures in order to perform their cellular functions (Cruz & Westhof, 2009 ▶; Novikova *et al.*, 2012 ▶; Westhof & Romby, 2010 ▶). These discoveries serve to further emphasize the importance of nucleic acid structure determination. Moreover, given their intrinsic properties, nucleic acids are also good targets for structure prediction *de novo* or by homology modeling. New computational approaches and software are being developed to create reliable three-dimensional models for medium-to-large nucleic acids even in the absence of experimental data. These models can help to expand the size of the nucleic acid structural databases and to increase the use of MR. Owing to this and the other factors discussed above, we expect that the rate of nucleic acid structures solved by MR in the future will be comparable to, if not higher than, that of protein structures.

## Figures and Tables

**Table 1:** Different MR strategies for nucleic acids of different lengths

	Short	Medium	Long
Size (nt)	<30–40	40–200	>200
Typical secondary/tertiary structure	Hairpins	Combinations of hairpins	Complex tertiary structures
Availability of experimental models† (%)	83.5	13.1	3.4
Identification of structural homologues	Based on size, independently of sequence	By structure-similarity algorithm (sequence covariation)	By structure-similarity algorithm (sequence covariation)
Strategies to improve MR success	Generally unnecessary	Pruning bases/bases and sugars	Pruning bases/bases and sugars
		Deletion of loops and junctions	Using only selected domains
			Supporting the MR search using preliminary experimental phases
Limitations of MR using experimental models	Internal helical symmetry	R.m.s.d.‡ up to 2–3 Å	R.m.s.d.‡ up to 2–3 Å
Reference models in the absence of experimental data	Ideal helices modeled manually	Combinations of ideal helices modeled manually or	Combinations of ideal helices modeled manually (rare) or
		Three-dimensional motifs modeled *de novo* or	Three-dimensional motifs modeled *de novo* or
		Homology models	Homology models
Limitations of MR using *in silico* designed models	Internal helical symmetry	Difficulty in assigning small helical domains	Difficulty in assigning small helical domains
		R.m.s.d.‡ up to 1–1.5 Å	R.m.s.d.‡ up to 1–1.5 Å
References	Baikalov & Dickerson (1998 ▶)	Scott (2012 ▶)	Humphris-Narayanan & Pyle (2012 ▶), Marcia & Pyle (2012 ▶) and this work

† Indicates the percentage of X-ray structures of nucleic acids of the corresponding size (statistics drawn from the PDB on 19 February 2013).

‡ Root-mean-square deviation between the MR search model and the target structure.
